# Intercellular crosstalk in adult dental pulp is mediated by heparin-binding growth factors Pleiotrophin and Midkine

**DOI:** 10.1186/s12864-023-09265-w

**Published:** 2023-04-06

**Authors:** Natnicha Jiravejchakul, Gabriela L. Abe, Martin Loza, Soyoung Park, Ponpan Matangkasombut, Jun-Ichi Sasaki, Satoshi Imazato, Diego Diez, Daron M. Standley

**Affiliations:** 1 Department of Genome Informatics, Research Institute for Microbial Diseases, Osaka University, 3-1 Yamadaoka, Suita, 565-0871 Japan; 2 Department of Microbiology, Faculty of Science, Mahidol University, Bangkok, 10400 Thailand; 3 Department of Advanced Functional Materials Science, Osaka University Graduate School of Dentistry, 1-8 Yamadaoka, Suita, 565-0871 Japan; 4 Laboratory of Functional Analysis in silico, Human Genome Center, The Institute of Medical Science, The University of Tokyo, 4-6-1 Shirokane-dai, Minato-ku, Tokyo, 108- 8639 Japan; 5 Department of Systems Immunology, Immunology Frontier Research Center, Osaka University, 3-1 Yamadaoka, Suita, 565-0871 Japan; 6 Department of Dental Biomaterials, Osaka University Graduate School of Dentistry, 1-8 Yamadaoka, Suita, 565-0871 Japan; 7 Quantitative Immunology Research Unit, Immunology Frontier Research Center, Osaka University, 3-1 Yamadaoka, Suita, 565-0871 Japan

**Keywords:** Dental Pulp, Single-cell analysis, RNA-seq, Transcriptome, Fibroblasts, Pleiotrophin, Midkine, Homeostasis, Paracrine communication

## Abstract

**Background:**

In-depth knowledge of the cellular and molecular composition of dental pulp (DP) and the crosstalk between DP cells that drive tissue homeostasis are not well understood. To address these questions, we performed a comparative analysis of publicly available single-cell transcriptomes of healthy adult human DP to 5 other reference tissues: peripheral blood mononuclear cells, bone marrow, adipose tissue, lung, and skin.

**Results:**

Our analysis revealed that DP resident cells have a unique gene expression profile when compared to the reference tissues, and that DP fibroblasts are the main cell type contributing to this expression profile. Genes coding for pleiotrophin (*PTN*) and midkine (*MDK*), homologous heparin-binding growth-factors, possessed the highest differential expression levels in DP fibroblasts. In addition, we identified extensive crosstalk between DP fibroblasts and several other DP resident cells, including Schwann cells, mesenchymal stem cells and odontoblasts, mediated by *PTN* and *MDK*.

**Conclusions:**

DP fibroblasts emerge as unappreciated players in DP homeostasis, mainly through their crosstalk with glial cells. These findings suggest that fibroblast-derived growth factors possess major regulatory functions and thus have a potential role as dental therapeutic targets.

**Supplementary Information:**

The online version contains supplementary material available at 10.1186/s12864-023-09265-w.

## Introduction

Dental pulp (DP) is a soft tissue of ectomesenchymal origin, located within teeth, and surrounded by rigid dentine walls. Vascularization and innervation are supplied through the apical foramen, a narrow opening at the end of each dental root. This anatomical configuration often makes relatively minor inflammation result in severe pain and tissue necrosis, by strictly limiting tissue swelling and increasing internal pressure. If tissue vitality is preserved, then damaged tissue can be recovered by the regenerative abilities of DP. DP regenerates by recruiting the same cell types involved in homeostasis, and no new cell type that is unique to injury has been observed [1, 2]. Although DP tissue repair can be promoted by various dental treatments such as pulp capping, few interventions harness its regenerative properties. Currently, the composition of DP at the cellular and molecular levels, as well as the mechanisms by which DP resident cells maintain homeostasis, are not well understood.

Several studies have reported interactions among cells in DP, with special attention given to mesenchymal stem cells (MSCs) and their niches [3, 4]. It is now accepted that DP possesses two main MSCs niches, perivascular and neural, which participate in tissue development and regeneration [5]. Within these niches, mechanisms of self-regulation have been discovered. However, it is not clear how other resident and transient cells interact with these niches or participate in the regulation of overall tissue homeostasis.

Single-cell transcriptomics allows the quantification of gene expression in individual cells in a tissue sample. Recent analysis of human DP have identified cells involved in tissue development [1], delineated major cellular components of adult DP [6], and described enriched cell types under carious lesion conditions [7].

Here, we sought to characterize DP tissue at the cellular and molecular levels by integrating the data from all publicly available healthy human DP single-cell transcriptome datasets. As a reference, we integrated data from five different human tissues of healthy adults. We hypothesized that the gene expression patterns in DP cells would be distinct from those in the corresponding cell types in reference tissues. We further reasoned that, if such unique phenotypes were observed, they would help shed light on the biological processes responsible for DP tissue homeostasis. Such insight may further inform the development of cellular and molecular-based treatment strategies in dentistry.

## Results

### DP resident fibroblasts exhibit unique gene expression profiles

**Fig. 1:**
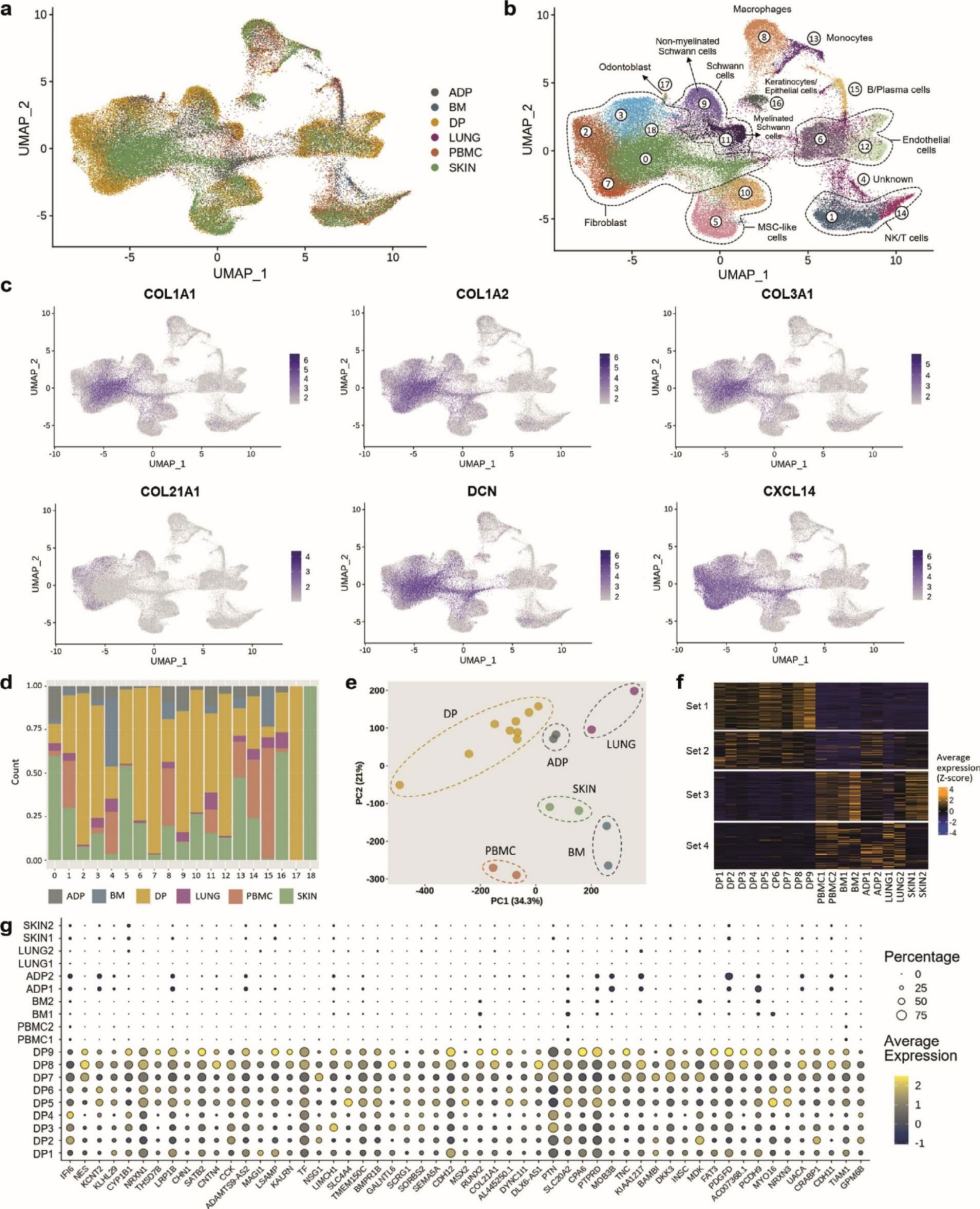
**scRNA-seq analysis of human dental pulp and reference tissues from healthy adults.** (**a**) UMAP visualization of all integrated tissues, cell clusters are colored by tissue of origin. (**b**) UMAP visualization of unbiased cluster classification identifying 19 cell clusters with cell type annotation. (**c**) Feature plots of fibroblast markers identifying the cell population within clusters 0, 2, 3, 7 and 18. (**d**) Bar plot of the fraction of cells that compose each cluster shown in **c**, colored by tissue of origin. (**e**) Principal component analysis plot of fibroblasts performed using average gene expression for each sample. (**f**) Heatmap of the top 500 differentially expressed genes that contributed to PC1 and PC2 in **e**, set 1 shows upregulated genes in DP fibroblasts. (**g**) Dot plot of curated genes of interest retrieved from heatmap set 1, the complete list of genes is provided in Supplementary Table 1

The cell composition of and transcriptomic signatures within DP tissues were investigated by integrating nine DP single-cell RNA sequencing datasets with ten datasets from five reference tissues: peripheral blood mononuclear cells (PBMC), bone marrow (BM), adipose tissue (ADP), lung (LUNG), and skin (SKIN). We selected these tissues because they contain representatives of the major cell types populating the DP [5, 8]. After applying all QC filters, the resulting dataset contained 109,554 cells, and 33,401 genes, including 48,659 cells from DP and 60,895 from other tissues (Fig. 1a). Clustering identified nineteen distinct cell clusters which were further annotated by cell type (Fig. 1b) (Supplementary Fig. 1a) using established markers from the literature along with CellTypist encyclopedia [9] (Supplementary Fig. 1b). Overall, fibroblasts which were identified by the expression of markers *COL1A1*, *COL1A2*, *COL3A1*, *DCN*, and *CXCL14* (Fig. 1c), were the most abundant cell type. We found a very small cluster of *MKI67* + proliferating fibroblasts (pro-fibroblasts) (Cluster18) that was present only in skin tissue (Fig. 1d). Schwann cells (Schw), which consisted of myelinated Schw (*MBP*+) and non-myelinated Schw (*GFRA3*+) were identified by the expression of *SOX10* [10, 11] (Supplementary Fig. 2). DP was enriched in both fibroblasts and Schw clusters (Fig. 1d). In addition, a small cluster of odontoblasts was identified by the expression of *DMP1* (Fig. 1b) (Supplementary Fig. 3). Other cells identified included immune cells (cluster 8, 13, 15, 1, and 14), endothelial cells (clusters 6 and 12), epithelial cells and keratinocytes (cluster 16). Finally, two clusters (5 and 10) with markers compatible with perivascular cells (*CSPG4*) [12], including vascular smooth muscle cells (*ACTA2*, *TAGLN*, and *TPM2*) [13, 14], and mesenchymal stem cells (*ACTA2*, *FRZB*, *NOTCH3*, and *MYH11*) [6, 12] were annotated as MSC-like cells (Supplementary Fig. 4).

To identify tissue-specific features, we performed PCA for each cell cluster in Fig. 1b using average expression profiles (pseudo-bulk) for each sample. The corresponding plots showing the pseudo-bulk sample distribution in the first 2 principal components (PCs) of all clusters are shown in Supplementary Fig. 5. The PCA plots indicated that DP fibroblasts (Cluster 0, 2, 3, and 7) and Schwann cells (Cluster 9 and 11) were well separated from those of other reference tissues, suggesting the existence of DP specific markers driving this separation (Supplementary Fig. 5).

In order to understand the gene expression signatures unique to DP fibroblasts, we performed PCA on pseudo-bulk signatures using cells in the fibroblast clusters (0, 2, 3 and 7). For this analysis, proliferating fibroblasts (cluster 18) were not included, as they were found only in skin. The first two components (PC1 and PC2) showed separation between fibroblasts from DP compared to fibroblasts from other tissues (Fig. 1e). Then, the top 500 genes contributing to PC1 and PC2 were obtained and used to construct a heatmap (Fig. 1f). The heatmap highlighted a group of genes (set 1) as being upregulated in DP fibroblasts while downregulated in fibroblasts of other reference tissues. We further curated genes from the heatmap set 1 to construct a dot plot with genes of interest showing the highest average expression in DP (Fig. 1g) (a complete list of genes retrieved from heatmap set 1 is provided in Supplementary Table 1). Taken together, our analyses revealed that DP have a unique gene expression profile when compared to the five reference tissues and that DP fibroblasts are the main contributors to these features.

### Heparin-binding growth factors pleiotrophin (PTN) and midkine (MDK) are highly expressed in DP resident fibroblasts

**Fig. 2:**
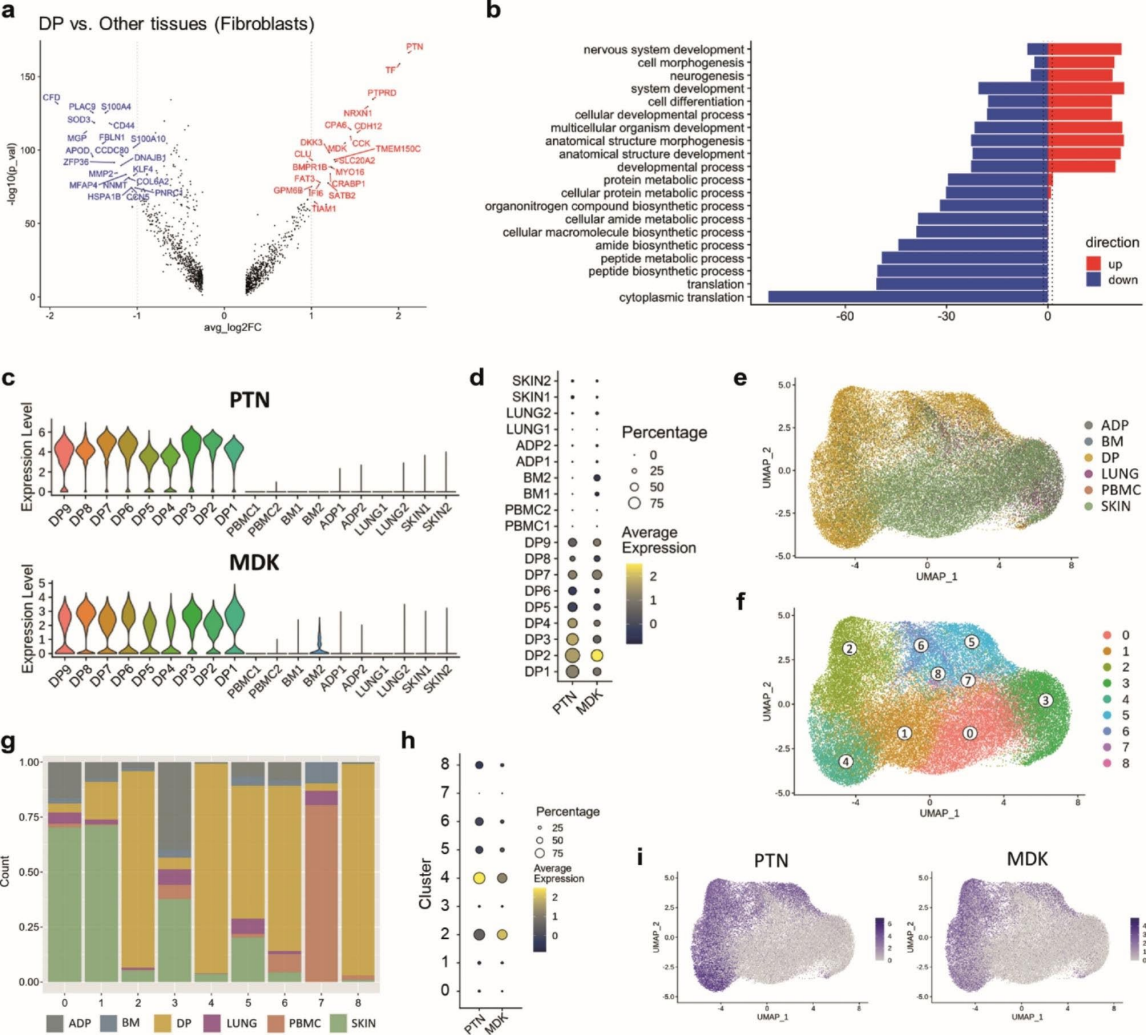
**Dental pulp fibroblasts versus those from reference tissues.** (**a**) *PTN* and *MDK* are differentially expressed genes in DP fibroblast versus reference tissue fibroblasts. (**b**) Gene ontology enrichment analysis using the DE genes from **a** identified significantly upregulated Biological Processes in DP fibroblasts. (**c**) Violin plots of *PTN* (upper) and *MDK* (lower) expression levels in fibroblasts of all tissues. (**d**) Dot plot showing percentage and average expression of *PTN* and *MDK* of fibroblasts of all tissues. (**e**) UMAP visualization of fibroblasts from all tissues integrated and colored by tissue of origin. (**f**) Unbiased classification identifying 9 clusters of fibroblast sup-populations. (**g**) Bar plot of the fraction of cells that compose each cluster shown in **f**, colored by tissue of origin. (**h**) Expression of *PTN* (upper) and *MDK* (lower) projected onto the UMAP plot of integrated fibroblasts. (**i**) Dot plot showing percentage and average expression of *PTN* and *MDK* by clusters of fibroblasts in **f**

To gain insight into DP fibroblast specific genes at single-cell resolution, we next compared the differential gene expression between DP fibroblasts and those from reference tissues. The volcano plot in Fig. 2a highlights genes with large fold changes that are also statistically significant. The eight most elevated genes in DP fibroblasts were *PTN*, *TF*, *PTPRD*, *NRXN1*, *CDH12*, *CCK*, *CPA6*, and *MDK* (Fig. 2a), consistent with the genes retrieved from PCA pseudo-bulk analysis shown in Fig. 1g. We next performed gene set enrichment analysis to investigate which gene ontology (GO) terms were under- or over-represented in these genes. The top GO terms in the Biological Process ontology were: “nervous system development”, “cell morphogenesis”, “neurogenesis”, “system development” and “cell differentiation” (Fig. 2b).

Within the DE genes in Fig. 2a, *PTN*–the most upregulated gene in DP fibroblasts–and *MDK*, are homologs coding for Pleiotrophin and Midkine, respectively. *PTN* and *MDK* are members of a distinct family of secreted heparin-binding growth factors that have been implicated in various biological processes, from development to inflammation to tumorigenesis [15]. We found that *PTN* and *MDK* were highly expressed in all nine DP datasets, while their levels were extremely low in all ten datasets from the other five reference tissues (Fig. 2c, d).

To further investigate the unique contribution of *PTN* and *MDK* to DP fibroblasts, fibroblast clusters from all six tissues were independently integrated, and UMAP coordinates and clusters were recalculated, resulting in nine subpopulations (Fig. 2e, f). Fibroblast clusters 2, 4, 5, 6 and 8 were enriched significantly in DP tissues (Fig. 2g). As expected, *PTN* and *MDK* were expressed only in these DP-resident fibroblast clusters (Fig. 2h, i), confirming that *PTN* and *MDK* are the key genes distinguishing the DP fibroblasts from the reference tissue fibroblasts.

### Analysis of DP cell populations and PTN- and MDK-mediated intercellular communication networks

**Fig. 3:**
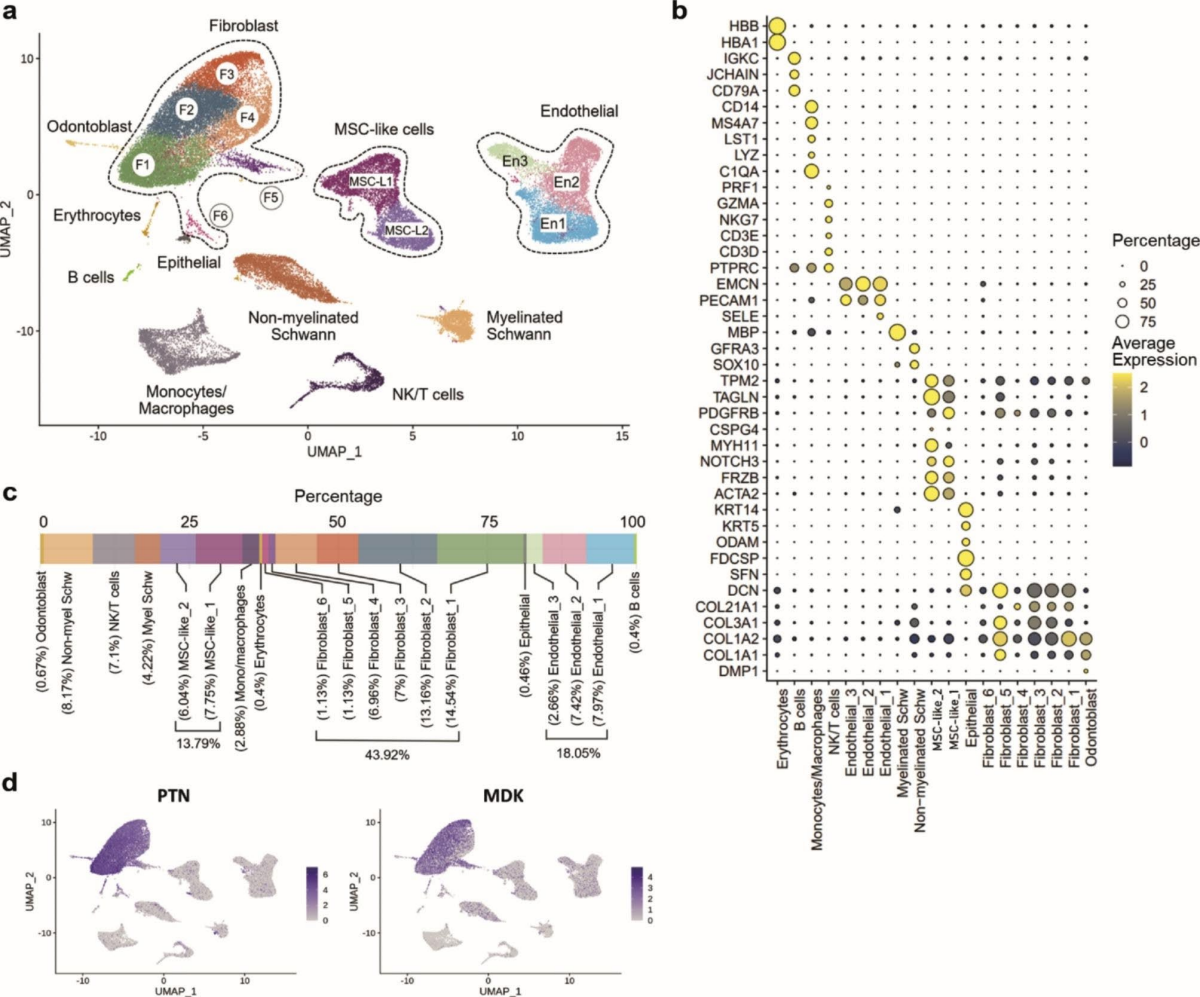
**ScRNA-seq analysis of integrated human dental pulp tissues.** (**a**) UMAP visualization of integrated DP datasets. (**b**) Expression levels of known cell type markers used for the cluster annotation in **a**. (**c**) Percentage of cell types that compose the healthy adult human DP. (**d**) Expression of *PTN* (left) and *MDK* (right) projected onto the UMAP plot of integrated dental pulp tissues, fibroblasts highly express *PTN* and *MDK*.

To understand the biological relevance of *PTN* and *MDK* in the DP environment, we next ignored the reference tissues and turned our attention exclusively to the DP datasets. Data integration of all nine healthy DP datasets retrieved from three different studies (Supplementary Table 2) was performed. Unbiased clustering predicted nineteen different clusters, which were further grouped into eleven cell types (Fig. 3a) using known markers indicated in Fig. 3b. Our results revealed that fibroblasts were the largest cell population in the dental pulp (43.92%), followed by endothelial cells (18.05%), MSCs (13.79%) and Schwann cells (12.39%) when combining non-myelinated and myelinated cells. While B cells and erythrocytes were the smallest cell cluster (0.4%) (Fig. 3c). As expected, elevation of *PTN* and *MDK* expression was found in clusters annotated as fibroblasts (Fig. 3d).

**Fig. 4:**
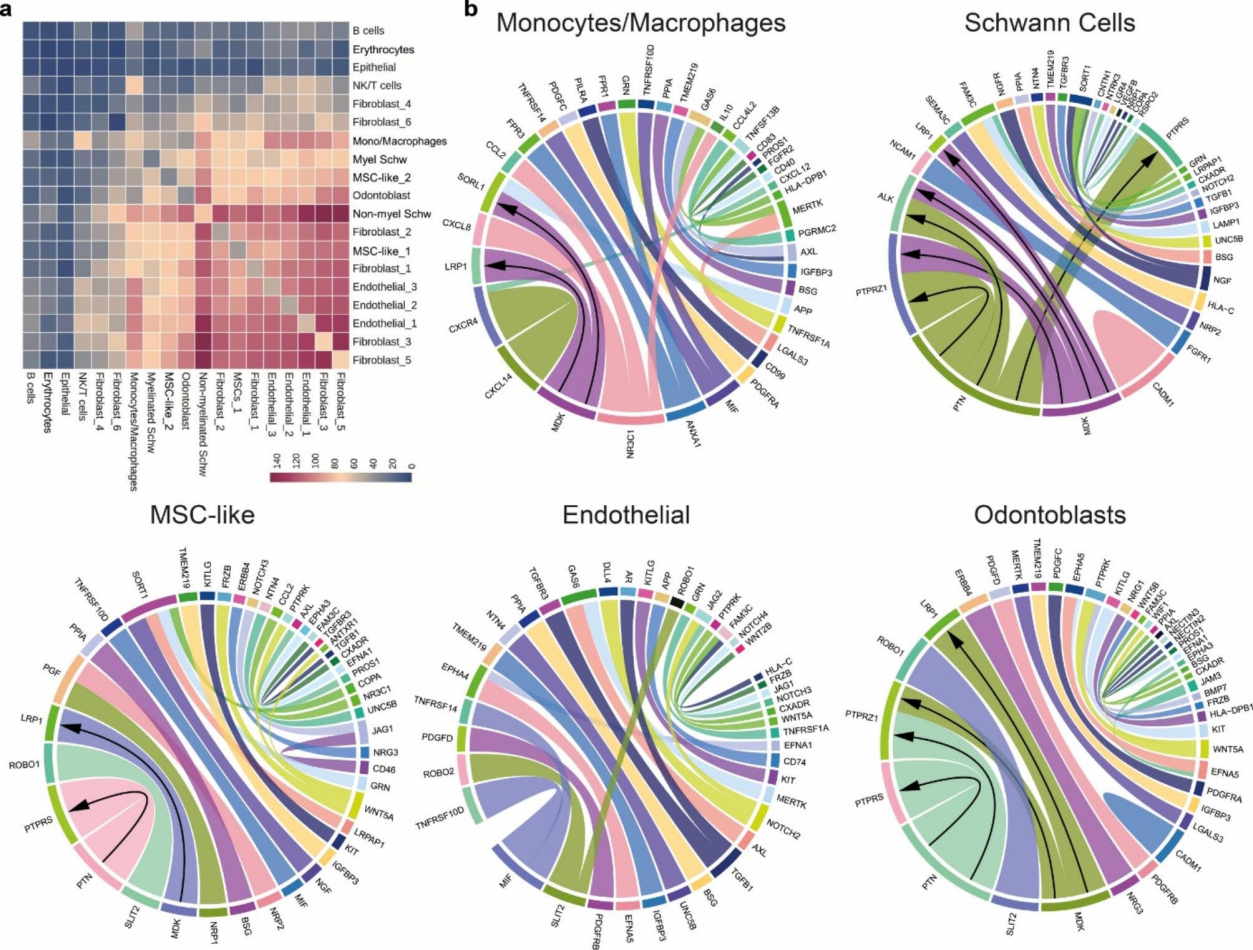
**CellPhoneDB analysis predicted *PTN *and *MDK *interactions in the dental pulp.** (**a**) Heatmap showing the total number of statistically significant predictions of cell-cell interactions. (**b**) Chord diagrams of the top 25 ligand-receptor pairs between fibroblasts and their corresponding cell types. Line thickness represents an interaction score obtained from fibroblast clusters involved in the interaction. Arrows indicate *PTN* and *MDK* on fibroblasts and their predicted interacting partners on the predicted target cell type

Since *PTN* and *MDK* are secreted heparin-binding molecules, we sought to investigate potential target cells based on the mRNA expression of their known receptors using CellPhoneDB[16]. Our analysis revealed a high number of overall interactions between fibroblasts and non-myelinated Schwann cells, MSCs, endothelial cells and odontoblasts (Fig. 4a), including interactions independent of *PTN* or *MDK*. To illustrate specific ligand-receptor pairs contributing to these interactions between fibroblasts and other cell types, chord diagrams were constructed using the top twenty-five interacting pairs (Fig. 4b). It is shown that *PTN* and *MDK* were the top molecules mediating major communication between fibroblasts and non-myelinated Schwann cells, MSCs and odontoblasts, but not endothelial cells (Fig. 4b, Supplementary Fig. 6). In addition, even though fibroblast interacted with several DP cells via *PTN* and *MDK*, the prediction for their receptors varied depending on the cell type (Fig. 4b, Supplementary Fig. 6).

*PTN* and *MDK* bind both heparin and heparan sulfate (HS). These are linear polysaccharides attached to cell surfaces by one or more anchoring proteins. In order to further support the role of *PTN*- and *MDK*-mediated communication between DP fibroblasts and various other DP resident cells, we next investigated the expression levels of genes encoding major HS anchor proteins. We found *BGN*, *GPC3*, *SDC2* were expressed on cells that also expressed high levels of receptors for *PTN* and *MDK* (Supplementary Fig. 7a). In addition, we determined the expression of genes involved in the biosynthesis of HS itself (Supplementary Fig. 7b). Taken together, these results support a model where *PTN* and *MDK* are produced by DP-resident fibroblasts and exert a paracrine effect on several types of DP cells, most significantly Schwann cells and odontoblasts.

## Discussion

In order to characterize DP tissue homeostasis, we integrated all publicly available scRNA-seq data from healthy human DP and compared the expression profiles with several well-studied reference tissues. We hypothesized that, due to the unique functional constraints on DP cells, their gene expression patterns would be distinct from those of other tissues. Our analysis revealed that DP tissue indeed exhibited a unique gene expression profile in comparison to reference tissues and that DP resident fibroblasts are the source of these differences.

Other cellular components of the tissues studied here, such as endothelial cells and immune cells, did not show significant differences, suggesting a common behavior across those tissues. While fibroblasts are found throughout the human body, their function is dependent on local environment and context to support other resident cells and engage in tissue remodeling when necessary [17]. In our study, we evaluated data obtained from healthy adult DP tissues, and assumed a context of steady state and homeostasis. A total of six subpopulations of fibroblasts were identified, revealing a previously unappreciated diversity, suggesting that each distinct fibroblast subtype within DP may be involved in distinct functional roles. Characterization of these subpopulations is now necessary and particular attention should be placed on the identification of non-overlapping functions, as these may be used to develop new therapeutics.

Studies on single-cell analysis of human DP are few, and their methodologies differ both in sequencing platform and chemistry. To minimize batch effects in our analysis we selected only studies that used equivalent and most current methodology, ignoring those that used different platforms. However, our results largely agree with previously published data, regardless of their methodology. Yin et al. identified similar cell types as were observed here; a cluster labeled “pulp cells”–which can be regarded as fibroblasts given their expressed markers–showed an active communication network with other cells in the DP [18]. MSCs were found to be the main cluster interacting with fibroblasts; however, in addition to MSCs, we show that fibroblasts have significant interactions with Schwann cells, endothelial cells and odontoblasts. Our integrative approach, that included multiple datasets from the literature, yielded a greater number of cells than any individual study capturing additional significant interactions in the communication networks within cell clusters.

Our analysis identified several functional genes contributing to the unique expression profile of DP-resident fibroblasts: Transferrin (*TF*), reported to support tooth morphogenesis and dental cell differentiation [19]; Cadherin 12 (*CDH12*), which plays an important role in calcium-dependent cell adhesion and has been implicated in oral tumors [20]; Cholecystokinin (*CCK*), which regulates calcium transport in organ mineralization [21]. Notably, genes related to nervous system development and neurogenesis were differentially expressed, a finding reiterated by the GO analysis. Receptor-type tyrosine-protein phosphatase delta (*PTPRD*), identified as a critical growth suppressor in the central nervous system (*CNS*) [22]; Neurexin 1 (*NRXN1*), a receptor found in CNS synapses that plays an important role in neural development [8]; Carboxypeptidase A6 (*CPA6*), involved in the processing of neuropeptides and reported to be upregulated in DP [23]. Taken together, these findings are consistent with the ectomesenchymal origin of DP.

The embryological development of craniofacial connective tissues involves neural crest-derived cells that acquired mesenchyme features, and thus are termed ectomesenchymal cells [24]. This neural component of DP tissues remains relevant after development. In adult tissue, peripheral axons comprise 40% of DP volume [25], are the basis for pain processing [26], and participate in immune response [27]. Although neurons cannot be isolated by the tissue digestion techniques applied in the sample processing for scRNA-seq, we were able to show that glial cells alone, myelinated and non-myelinated Schwann cells, comprise 12.39% of the DP cell population. It has been reported that sensory nerves recruit MSC through paracrine signaling [4], our analysis indicate that opposing mechanisms may emerge from DP-resident fibroblasts and glial cells through paracrine regulation.

Among the differentially expressed genes in DP-resident fibroblasts *PTN* appear as the top significant and *MDK* is its highly conserved homologue. Both *PTN* and *MDK* code for biochemically similar proteins that share 45% identity in amino acid sequence and exhibit strong binding to sulfated glycosaminoglycans, including heparin, heparan sulfate and chondroitin sulfate [15, 17]. Recently, Zhang and co-workers examined the role of *PTN* in DP stem cell maintenance, and, using RNA interference in cultured DP, showed that *PTN* protects DP stem cells from senescence [28]. *MDK* is essential in tooth development, participating in epithelial-mesenchymal interactions, where it is secreted by cells of the dental papilla mesenchyme and captured by epithelial cells [29]. This mechanism was further elucidated in a later report, using dental papilla cells in vitro, where *MDK* could promote cell differentiation into odontoblast-like cells via the modulation of *dspp* [30]. However, the role of *MDK* in the adult healthy DP tissue homeostasis is largely unknown. High expression of *MDK* has been previously identified by microarray screening, but expression levels dropped dramatically when compared to caries conditions [31]. Together, these studies indicate that *MDK* is involved in cell proliferation and differentiation but the circumstances leading to the presence or absence of *MDK* are unclear.

Cell-cell communication predictions indicated that among the top 25 significant pairs, *PTN*/*PTPRZ1* may be especially relevant to understanding DP homeostasis. Upon receptor binding, *PTN* inactivates the intrinsic tyrosine phosphatase activity of *PTPRZ1* [32], affecting several downstream targets that are highly regulated by phosphorylation. These include β-catenin and Fyn kinase [33], which are essential to cell proliferation and differentiation, to cell adhesion and cell motility, and regulation of apoptosis. Similarly, *MDK* binding to *PTPRZ1* is predicted to have analogous effects given their homology [15, 34]. It is known that primary cultures of DP stem cells from explanted adult DP shows a high proliferation rate in vitro [35], however, this is not observed in vivo where cell mitosis is undetectable in homeostasis [36, 37]. Our analysis revealed for the first time that the mechanisms for tissue homeostasis, through which DP controls its extraordinary proliferative and differentiation abilities, may involve *PTN*/*PTPRZ1* binding or their homologues.

As life expectancy has increased, the maintenance, repair, and regeneration of dental tissue has become ever more important. Here we identified *PTN* and *MDK* as previously unappreciated players in DP homeostasis. The extensive receptors for these proteins on Schwann cells, along with the ectomesenchymal origin of DP, imply extensive crosstalk between fibroblasts and nerve cells in the regulation of this tissue. A limitation of this work is that computational predictions reported here must be validated in vitro, which will further refine our understanding of intercellular crosstalk in dental pulp. There is a great need for synthetic molecules capable of targeting DP accurately and precisely. Once the putative mediators of intercellular crosstalk identified here are validated experimentally, a natural next step will be to screen small molecules that can target these proteins. Undoubtedly, the treatment of oral diseases will benefit from the ability to specifically target drugs to DP resident fibroblasts.

## Methods

### Dataset selection

FASTQ files of single-cell RNA sequencing (scRNA-seq) data were retrieved from published studies [1, 6, 7, 13, 38]. All datasets were generated by the Chromium single-cell gene expression platform from 10x Genomics 5’ V1 and 3’ (V 2 or V3) to avoid other confounding factors and technical biases. In total, we included nineteen datasets from six different healthy human tissues; nine DP (GSE185222, GSE161267, and GSE146123), two bone marrow (BM) (PRJEB37166), two adipose (ADP) (GSE155960), two lung (PRJEB52292), two skin (PRJNA754272) and two peripheral blood mononuclear cells (PBMC). The DP datasets deposited under the above accession numbers contained both healthy and carious DP data, as well as periodontal tissue data, however only data originated from healthy DP tissue were downloaded for analysis in this study. The two PBMC data were acquired from publicly available datasets at 10x Genomics (Supplementary Table 2).

### Data pre-processing and quality control

Raw FASTQ files of each dataset were processed with 10x Genomics Cell Ranger version 7.0.0 and mapped against the human reference genome GRCh38 version 2020-A (GENCODE v32/Ensembl 98). Count matrices were then processed with the Seurat package (version 4.1.1) [39] in R (version 4.2.0). Quality control was performed by excluding the cells expressing mitochondrial genes higher than 10% or those expressing lower than 200 genes or higher than 4,000 genes. Cells that expressed more than 3,500 genes were filtered out for the integration of nine dental pulp datasets. Genes that were detected in fewer than three cells were discarded. Data were normalized by the log normalization method and scaled using the NormalizeData and ScaleData functions in Seurat [39] to yield a total count per cell of 10,000.

### Data integration and visualization

Data was integrated by the Seurat integration pipeline [40] using 2,000 high variable gene features across all datasets. The top thirty components from principal component analysis (PCA) were used to calculate a Uniform Manifold Approximation and Projection (UMAP) dimensionality reduction, after which clusters were identified using Louvain algorithm [41] based on a shared nearest neighbor (SNN) in the FindCluster function. Each cluster was manually annotated using cell type markers from the literature and curated markers from CellTypist [9].

### Quantifying transcriptomic expression of clusters and pseudo-bulk data

Pseudo-bulk PCA was performed using average gene expression across all cells within a cluster. The union of the top 500 genes having the highest absolute values for PC1 and PC2 were retrieved. The average expressions of these genes were transformed into Z-scores and subsequently used to construct a heatmap comparing gene expression profiles in DP with those of the other five tissues. The PCA plots and heatmaps were constructed using ggplot2 [42] and ComplexHeatmap R packages [43].

### Differential gene expression and gene ontology (GO) analysis

Fibroblast clusters were subset from the integrated dataset of the six tissues. Differential gene expression (DE) analysis of fibroblasts in DP and in other tissues was performed by the non-parametric Wilcoxon rank sum test using the FindMarkers function in the Seurat pipeline with gene expression levels before integration. The DE results were visualized by a Volcano plot using the scmisc R package (version 0.8.0). GO enrichment analysis for Biological Processes ontology was performed with the list of DE genes with FDR < 0.05, separating up-regulated and down-regulated genes. This analysis was done using the goana function implemented in the limma package[44] and wrapped for single cell analysis in the scmisc package.

### Cell-cell interaction analysis

Interactions between cells were predicted based on the expression levels of ligands and their known interacting partners by CellPhoneDB [16]. A heatmap showing potential interactions between cell types was constructed using the default visualizing function of CellPhoneDB. Dot plots showing the interacting pairs and the levels ligand-receptors expression were produced using the scmisc package. Significant means of ligand-receptor interactions from fibroblast clusters were summed to obtain a total interaction score between fibroblast and other cell types. The top 25 interactions were selected to create an adjacency matrix using the igraph R package [45] which was subsequently used to plot the chord diagrams with the circlize [46] package in R.

## Electronic supplementary material

Below is the link to the electronic supplementary material.

**Additional file 1: Supplementary Table 1**.

**Additional file 2: Supplementary Fig. 1**.

**Additional file 3: Supplementary Fig. 2**.

**Additional file 4: Supplementary Fig. 3**.

**Additional file 5: Supplementary Fig. 4**.

**Additional file 6: Supplementary Fig. 5**.

**Additional file 7: Supplementary Fig. 6**.

**Additional file 8: Supplementary Fig. 7**.

**Additional file 9: Supplementary Table 2**.

## Data Availability

Code availability: Code to reproduce the analyses described in this manuscript can be accessed via: https://github.com/NatnichaJira/DentalPulp Database links: GSE161267 (https://www.ncbi.nlm.nih.gov/geo/query/acc.cgi?acc=GSE185222); GSE161267 (https://www.ncbi.nlm.nih.gov/geo/query/acc.cgi?acc=GSE161267); GSE146123 (https://www.ncbi.nlm.nih.gov/geo/query/acc.cgi?acc=GSE146123); 10X Genomics (https://www.10xgenomics.com/resources/datasets); PRJEB37166 (https://www.ebi.ac.uk/ena/browser/view/PRJEB37166); GSE155960 (https://www.ncbi.nlm.nih.gov/geo/query/acc.cgi?acc=GSE155960); PRJEB52292 (https://www.ebi.ac.uk/ena/browser/view/PRJEB52292); PRJNA754272 (https://www.ebi.ac.uk/ena/browser/view/PRJNA754272).
