# Supplementary material for: Intercellular crosstalk in adult dental pulp is mediated by heparin-binding growth factors Pleiotrophin and Midkine
Source: BMC Genomics. 2023 Apr 6;24:184. doi: 10.1186/s12864-023-09265-w (PMC10077760; doi:10.1186/s12864-023-09265-w)
Supplement: Supplementary file 1 — Additional file 1: Supplementary Table 1. [file 12864_2023_9265_MOESM1_ESM.pdf]

Supplementary table 1

ABCA6	AC007368.1	AC022874.1	AC233723.1	ADAMTS9-AS2	ADAM12	AGAP1	AKAP12
AL445250.1	ANTXR1	APP	ARHGAP24	ARL15	BAMBI	BCAS3	BMPR1B
CACNB2	CALN1	CASC15	CBLB	CCK	CDH11	CDH12	CDK14
CHN1	CHRM3	CHSY3	CLU	CNTN4	COL21A1	COL5A3	DDX17
DKK3	DLC1	DLG2	DLX5	DLX6-AS1	DOCK4	DST	DYNC1I1
ECHDC2	EFNA5	EML4	ENOX1	EPS8	ERBIN	EXT1	FAM13A
FAT3	FBXL7	FRMD4A	FTX	GALNTL6	GAS7	GHR	GJA1
GPM6B	HMGN3	ID1	ID3	IFI44L	IFI6	IGFBP2	INSC
ISG15	ITM2C	IVNS1ABP	JMJD1C	KALRN	KAZN	KCNT2	KHDRBS2
KIAA1217	KIT	KLHL29	LARGE1	LIMCH1	LINC01060	LINC01515	LPP
LRP1B	LSAMP	MAGI1	MAGI2	MAML2	MAP4	MDK	MIR99AHG
MOB3B	MRC2	MSX2	MYO16	NAV2	NAV3	NES	NRG3
NRXN1	NRXN3	NSG1	NUDT4	N4BP2L2	PALLD	PARD3	PCDH9
PDE4B	PDGFD	PDLIM5	PHIP	PKP4	PLEKHA5	PLXDC2	PLXNA2
PNISR	PPP3CA	PRKD1	PRKG1	PTN	PTPRD	PYGL	RARRES1
RARRES2	RASGRF2	RGS3	ROBO1	RORA	ROR2	RUNX2	SATB2
SBF2	SCRG1	SEMA5A	SEPTIN11	SGCD	SHROOM3	SLC20A2	SLC4A4
SLIRP	SLIT2	SNED1	SORBS2	SPATS2L	SPPL2A	ST6GALNAC5	SYT1
TANC1	TENM3	TF	THSD7B	TIAM1	TNC	TNIK	TMBIM6
TMEM150C	TRPM7	TSC22D1	TTC28	UACA	UNC5B	UNC5C	WWOX
XIST	ZBTB20						
