# Supplementary figures and images for: Intercellular crosstalk in adult dental pulp is mediated by heparin-binding growth factors Pleiotrophin and Midkine
Source: BMC Genomics. 2023 Apr 6;24:184. doi: 10.1186/s12864-023-09265-w (PMC10077760; doi:10.1186/s12864-023-09265-w)

**A**

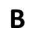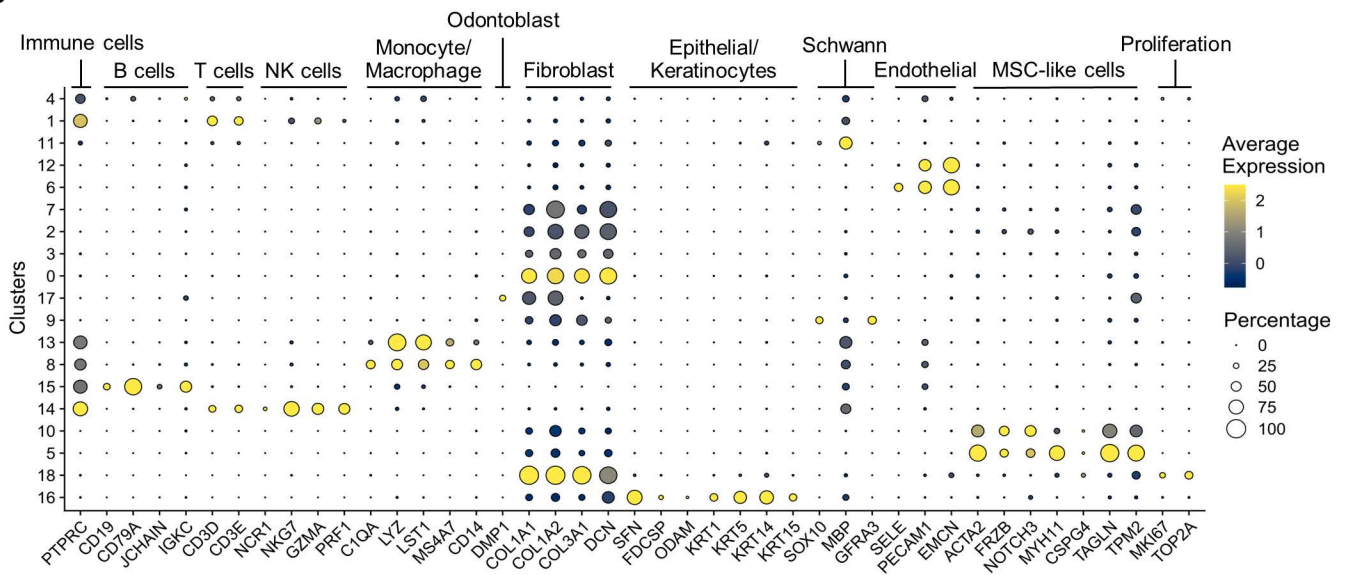

Supplement: Supplementary file 2 — Additional file 2: Supplementary Fig. 1. [file 12864_2023_9265_MOESM2_ESM.pdf]

# Supplementary Fig 2

**A**

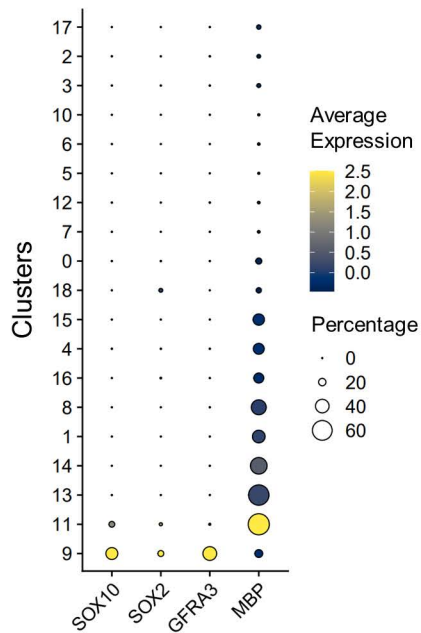

**B**

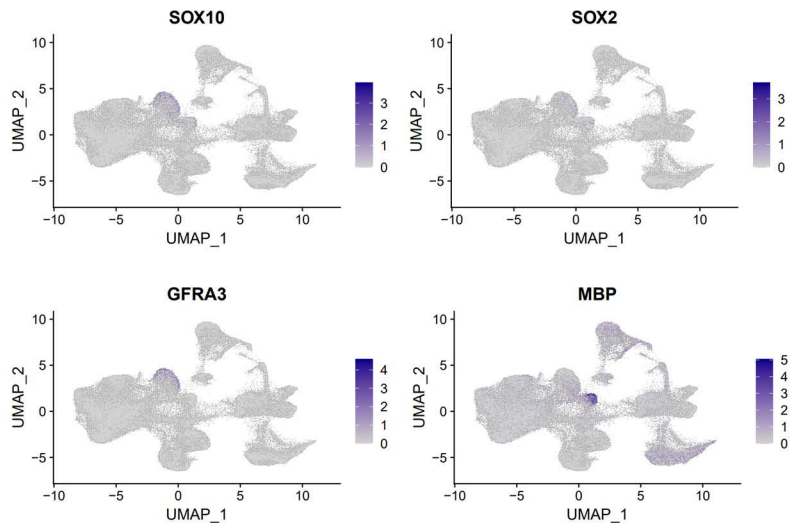

Supplement: Supplementary file 3 — Additional file 3: Supplementary Fig. 2. [file 12864_2023_9265_MOESM3_ESM.pdf]

# Supplementary Fig 3

**A**

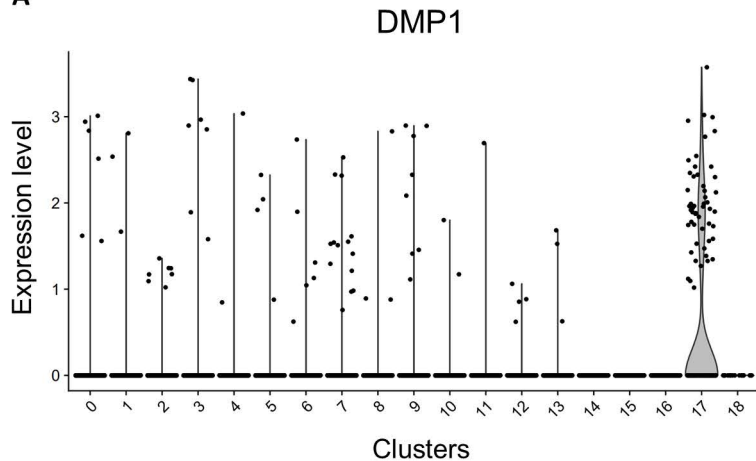

**B**

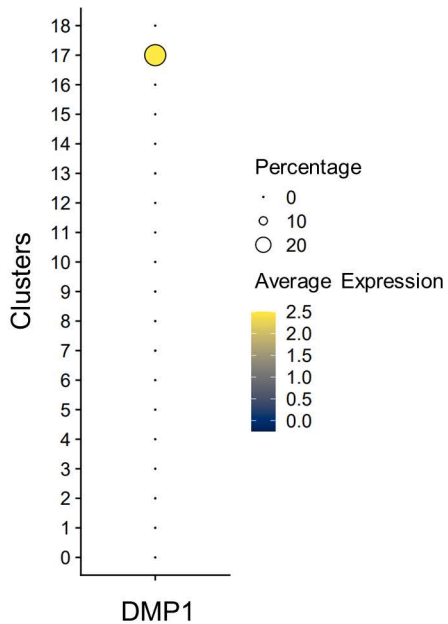

Supplement: Supplementary file 4 — Additional file 4: Supplementary Fig. 3. [file 12864_2023_9265_MOESM4_ESM.pdf]

Supplementary Fig 4

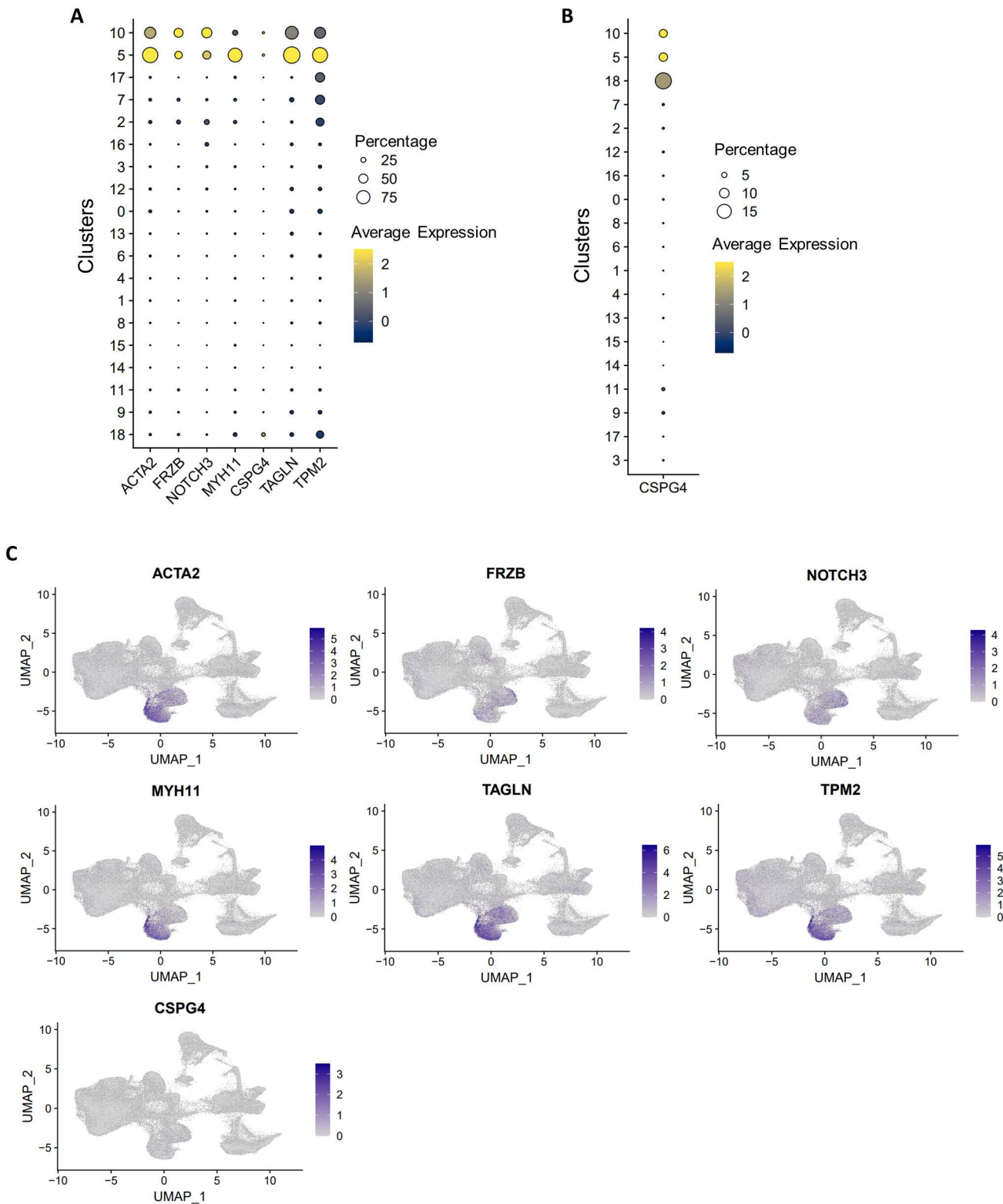

Supplement: Supplementary file 5 — Additional file 5: Supplementary Fig. 4. [file 12864_2023_9265_MOESM5_ESM.pdf]

Supplementary Fig 6

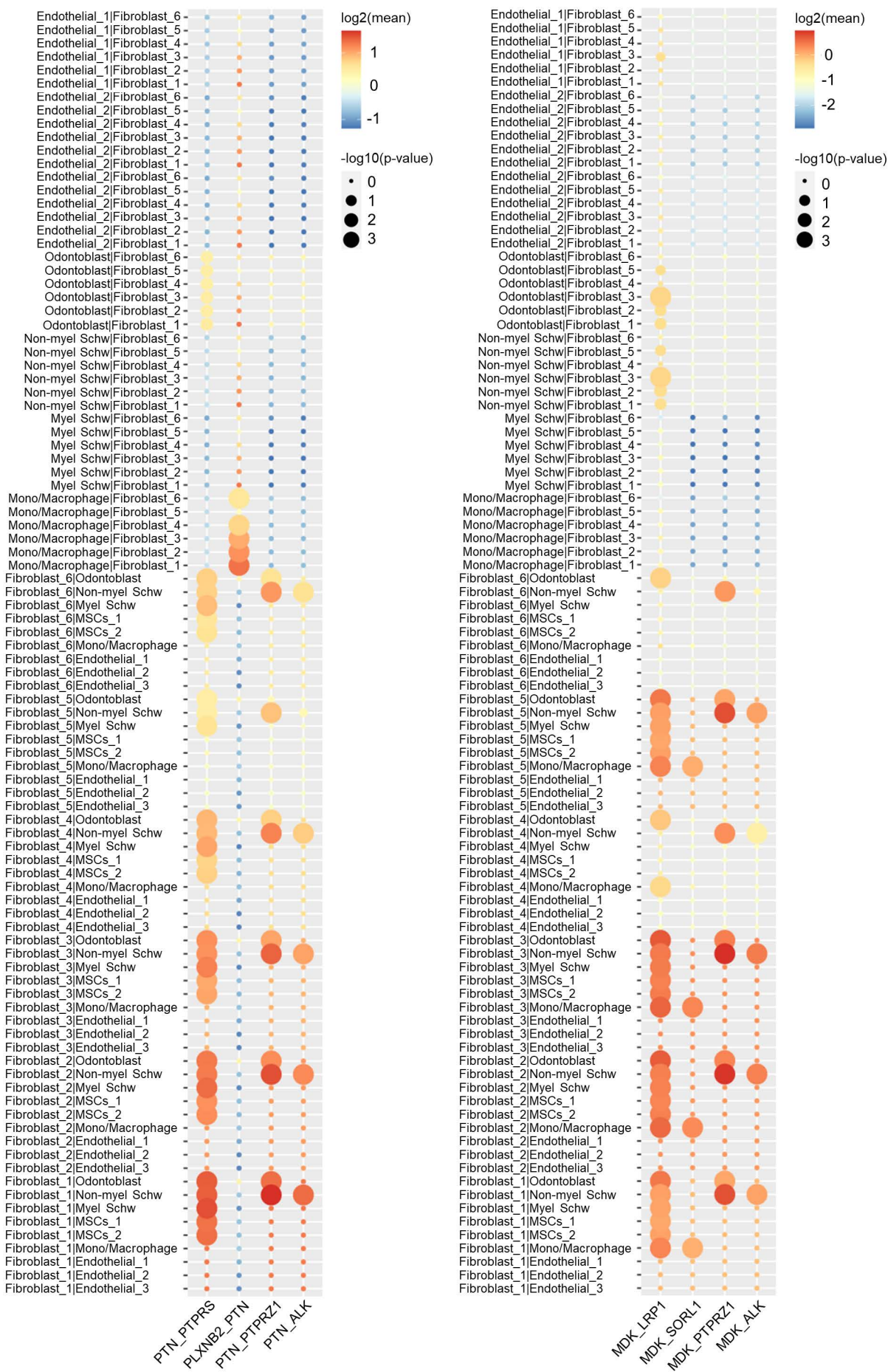

Supplement: Supplementary file 7 — Additional file 7: Supplementary Fig. 6. [file 12864_2023_9265_MOESM7_ESM.pdf]
