# Supplementary material for: Intercellular crosstalk in adult dental pulp is mediated by heparin-binding growth factors Pleiotrophin and Midkine
Source: BMC Genomics. 2023 Apr 6;24:184. doi: 10.1186/s12864-023-09265-w (PMC10077760; doi:10.1186/s12864-023-09265-w)
Supplement: Supplementary file 6 — Additional file 6: Supplementary Fig. 5. [file 12864_2023_9265_MOESM6_ESM.pdf]

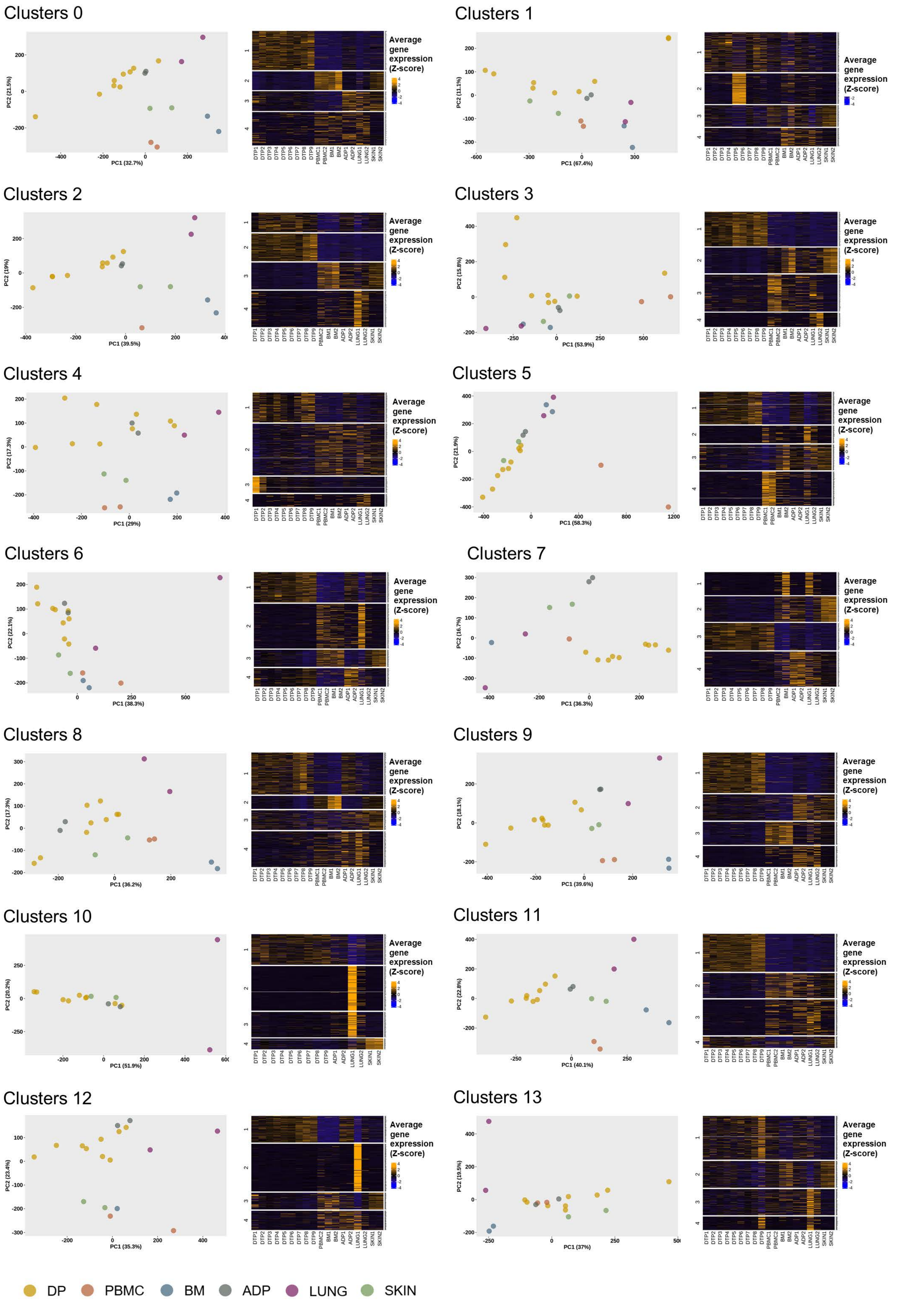
Clusters 0
PC1 (32.7%)
PC2 (21.5%)
Clusters 1
PC1 (67.4%)
PC2 (11.1%)
Clusters 2
PC1 (39.5%)
PC2 (19%)
Clusters 3
PC1 (53.9%)
PC2 (15.8%)
Clusters 4
PC1 (29%)
PC2 (17.3%)
Clusters 5
PC1 (58.3%)
PC2 (21.9%)
Clusters 6
PC1 (38.3%)
PC2 (22.1%)
Clusters 7
PC1 (36.3%)
PC2 (16.7%)
Clusters 8
PC1 (36.2%)
PC2 (17.3%)
Clusters 9
PC1 (39.6%)
PC2 (18.1%)
Clusters 10
PC1 (51.9%)
PC2 (20.2%)
Clusters 11
PC1 (40.1%)
PC2 (22.8%)
Clusters 12
PC1 (35.3%)
PC2 (23.4%)
Clusters 13
PC1 (37%)
PC2 (19.5%)
Average gene expression (Z-score)
DP
PBMC
BM
ADP
LUNG
SKIN

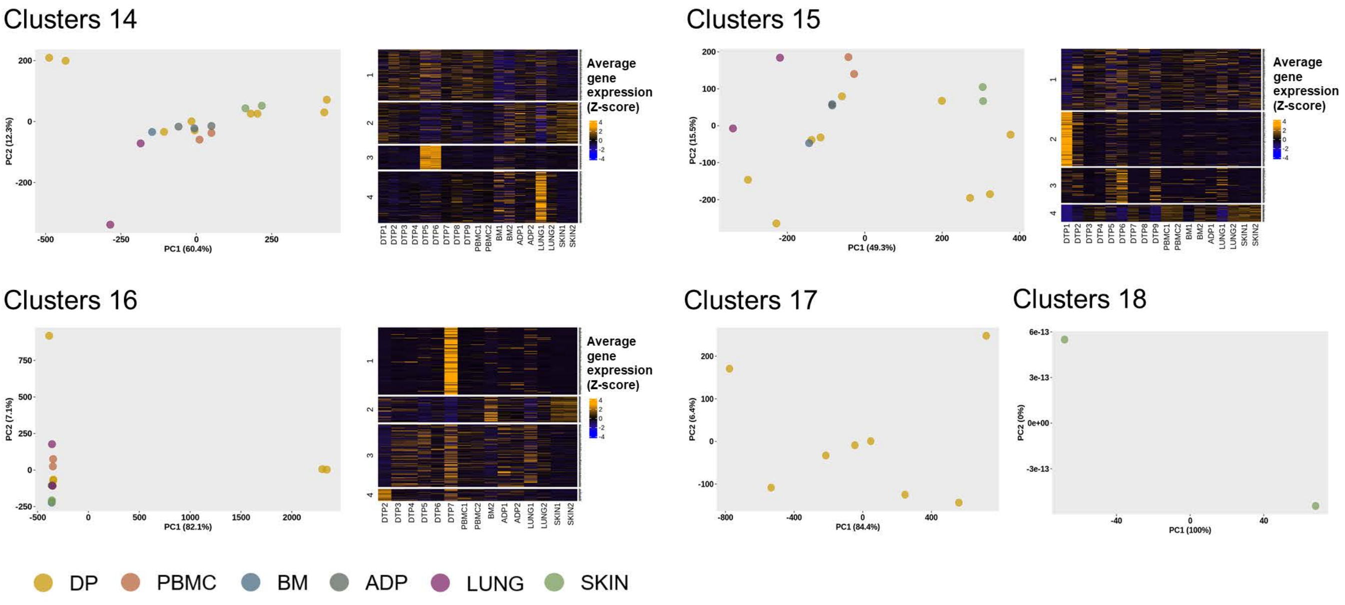

Supplementary Fig 5
