# Supplementary material for: Intercellular crosstalk in adult dental pulp is mediated by heparin-binding growth factors Pleiotrophin and Midkine
Source: BMC Genomics. 2023 Apr 6;24:184. doi: 10.1186/s12864-023-09265-w (PMC10077760; doi:10.1186/s12864-023-09265-w)
Supplement: Supplementary file 9 — Additional file 9: Supplementary Table 2. [file 12864_2023_9265_MOESM9_ESM.pdf]

Supplementary Table 2

Study/Project	Source	Platform	Chemistry solution	Tissue	Number of datasets	Run access/Sample ID	Sample description
GSE185222	NCBI	10x Genomics	3' V2	DP	1 (DP1)	GSM5608427	Upper third molar, sound, 21yo
GSE161267	NCBI	10x Genomics	3' V2	DP	5 (DP2-6)	GSM4998457-61	Upper third molars, sound, 18-35yo
GSE146123	NCBI	10x Genomics	3' V3	DP	3 (DP7-9)	GSM4365607-08, GSM4365602	Sound adult molars, age unknown
10x Genomics	10x Genomics*	10x Genomics	3' V2	PBMC	2	1K PBMC, 8K PBMC	PBMC from healthy adult donor
PRJEB37166	ENA	10x Genomics	3' V2	BM	2	ERR7363152, ERR7363153	Healthy adult bone marrow
GSE155960	NCBI	10x Genomics	3' V3	ADP	2	GSM4717152, GSM4717154	Adipose cells from healthy lean donors
PRJEB52292	ENA	10x Genomics	5' V1	LUNG	2	ERR9588945, ERR9588946	Healthy upper lobe of lung and bronchus
PRJNA754272	NCBI	10x Genomics	3' V2	SKIN	2	SRR15440581, SRR15440583	Skin from healthy adult (<30yo)

*Publicly available datasets from 10x Genomics
