# Supplementary material for: Intercellular crosstalk in adult dental pulp is mediated by heparin-binding growth factors Pleiotrophin and Midkine
Source: BMC Genomics. 2023 Apr 6;24:184. doi: 10.1186/s12864-023-09265-w (PMC10077760; doi:10.1186/s12864-023-09265-w)
Supplement: Supplementary file 8 — Additional file 8: Supplementary Fig. 7. [file 12864_2023_9265_MOESM8_ESM.pdf]

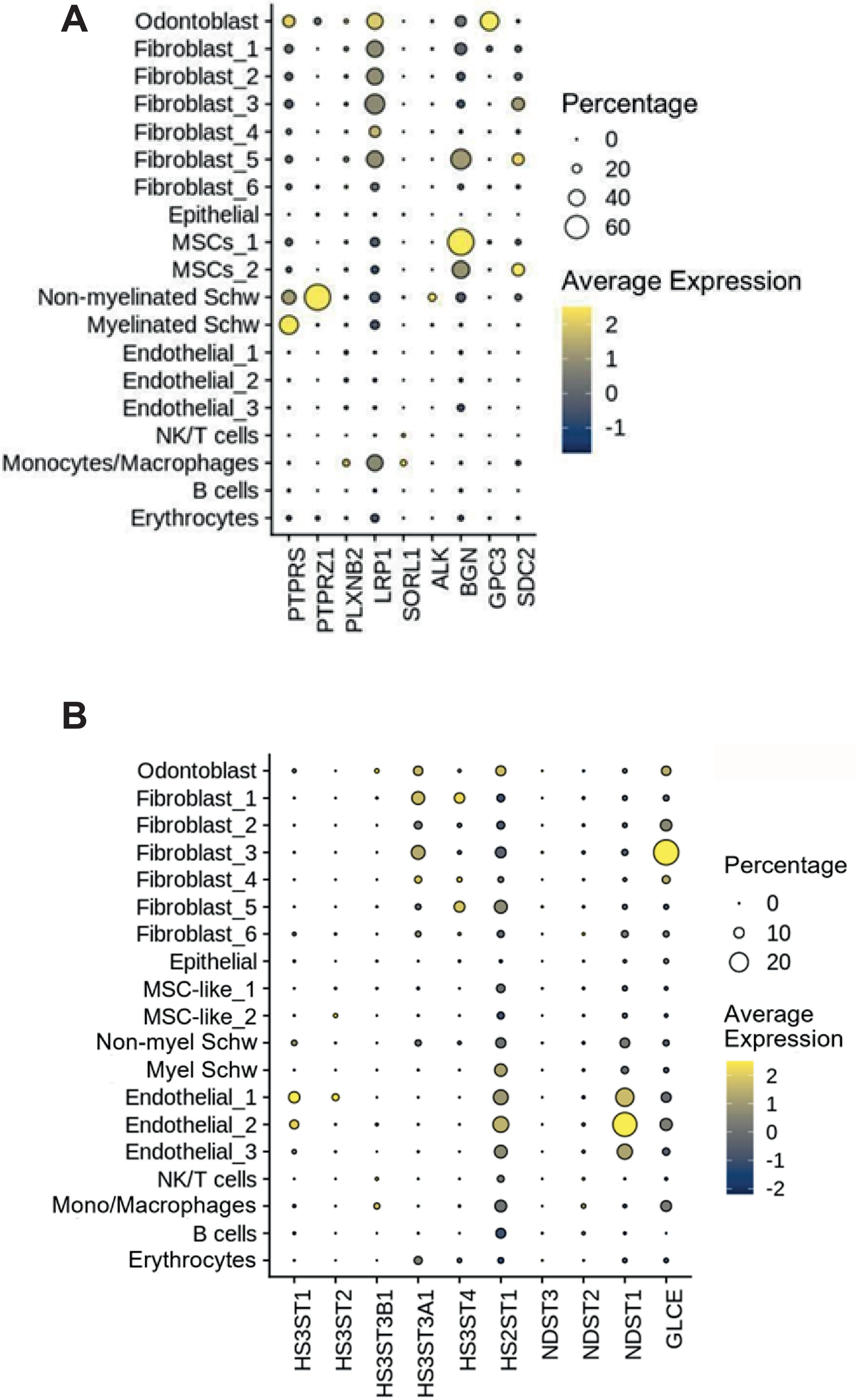
A
Odontoblast
Fibroblast_1
Fibroblast_2
Fibroblast_3
Fibroblast_4
Fibroblast_5
Fibroblast_6
Epithelial
MSCs_1
MSCs_2
Non-myelinated Schw
Myelinated Schw
Endothelial_1
Endothelial_2
Endothelial_3
NK/T cells
Monocytes/Macrophages
B cells
Erythrocytes
PTPRS
PTPRZ1
PLXNB2
LRP1
SORL1
ALK
BGN
GPC3
SDC2
Percentage
0
20
40
60
Average Expression
2
1
0
-1
B
Odontoblast
Fibroblast_1
Fibroblast_2
Fibroblast_3
Fibroblast_4
Fibroblast_5
Fibroblast_6
Epithelial
MSC-like_1
MSC-like_2
Non-myel Schw
Myel Schw
Endothelial_1
Endothelial_2
Endothelial_3
NK/T cells
Mono/Macrophages
B cells
Erythrocytes
HS3ST1
HS3ST2
HS3ST3B1
HS3ST3A1
HS3ST4
HS2ST1
NDST3
NDST2
NDST1
GLCE
Percentage
0
10
20
Average Expression
2
1
0
-1
-2
